# Proposal for a Histological Staging System of Mammary Carcinomas in Dogs and Cats. Part 2: Feline Mammary Carcinomas

**DOI:** 10.3389/fvets.2019.00387

**Published:** 2019-11-07

**Authors:** Florian Chocteau, Marie-Mélanie Boulay, Fanny Besnard, Germain Valeau, Delphine Loussouarn, Frédérique Nguyen

**Affiliations:** ^1^AMaROC (Animal Cancers, Models for Research in Comparative Oncology), Oniris, Nantes Atlantic College of Veterinary Medicine, Food Science and Engineering, Nantes, France; ^2^CRCINA, INSERM, Université d'Angers, Université de Nantes, Nantes, France; ^3^Department of Pathology, University Hospital, Nantes, France; ^4^Integrated Center for Oncology Nantes/Angers, Nantes, France

**Keywords:** cat, lymphovascular invasion, mammary carcinoma, pathologic nodal stage, pathologic tumor size, prognosis, stage, survival

## Abstract

**Background:** Feline mammary carcinomas (FMCs) are characterized by a high frequency of metastatic spread. The clinical TNM (Tumor, Node, Metastasis) system is used to describe local, regional, and distant tumor extent within the patient, but few publications confirmed its association with survival in cats with FMC. The purpose of this study was to determine if the histological staging system proposed for dogs in part 1 of this article had significant association with prognosis in cats.

**Materials and Methods:** This retrospective study included 395 female cats with a surgically removed mammary carcinoma, with a 2-year follow-up. Invasiveness (distinction between *in situ* and invasive FMCs), the pathologic tumor size (pT), lymphovascular invasion (LVI), and the pathologic nodal stage (pN) defined a 5-stage system: Stage 0 (FMCs *in situ*), Stage I (pT1, LVI–, pN0–pNX), Stage II (pT2, LVI–, pN0–pNX), Stage IIIA (pT1, LVI+ and/or pN+), and Stage IIIB (pT2, LVI+ and/or pN+), where pT1 was ≤20 mm, pT2 was >20 mm, and pNX corresponded to unsampled draining lymph node.

**Results:** Higher histological stages were associated with reduced disease-free interval, overall survival, and specific survival. For cancer-specific survival, by univariate analysis (*p* < 0.0001), median survival times and 1-year specific survival rates (1ySSR) were: stage 0 (1484 days; 1ySSR = 85%; *N* = 55; 14% of the cats), stage I (808 days; 1ySSR = 76%; *N* = 103; 26%), stage II (377 days; 1ySSR = 51%; *N* = 56; 14%), stage IIIA (448 days; 1ySSR = 60%; *N* = 83; 21%), and stage IIIB (207 days; 1ySSR = 29%; *N* = 98; 25%). The histological stages were also associated with specific survival by multivariate analysis (Hazard Ratio (HR) = 2.72 for stage IIIB, HR = 1.76 for stage IIIA, HR = 1.50 for stage II compared with stage I), independently of Progesterone Receptor expression (HR = 0.34 for PR+ compared with PR– FMCs) and tumor-associated inflammation (HR = 1.33 when moderate to severe compared with absent to mild).

**Conclusion:** A same histological staging system could be applied in dogs and cats with mammary carcinoma to refine prognosis assessment. In the near future, a preoperative complete tumor clinical staging and treatment based on the published standard of care should be performed in order to better validate the histological staging system here proposed.

## Introduction

Feline Mammary Carcinomas (FMCs) are among of the 3 most common malignancies in cats, with cutaneous/subcutaneous soft-tissue sarcomas, and malignant hemopathies (1–3). The annual incidence of mammary carcinomas was estimated to be 25 cases for 100,000 cats in an older report (4), however it was estimated in more recent series that the incidence of feline mammary tumors was 230/100,000 cats (1); as 80–90% of feline mammary tumors are malignant (5), the actual incidence of feline mammary carcinomas may be comprised between 184 and 207 per 100,000 cats, similar to canine mammary carcinomas.

As in dogs with invasive mammary carcinoma, a staging system can be used in cats to describe local, regional and distant cancer spread within the host. This TNM clinical staging system proposed by Owen^1^ and thereafter adapted by McNeill et al. (6), has been associated with significant prognostic value in studies with survival analyses (7–10) whereas only a few research projects assessed tumor extent using histological criteria. In 2002, Preziosi et al. have built a 3-stage histologically based system: stage 0 (FMCs *in situ*), stage I (invasive FMCs without nodal metastases nor lymphovascular invasion), and stage II (invasive FMCs with positive nodal stage and/or lymphovascular invasion) FMCs were associated with significantly decreasing overall survival probabilities in a cohort of 33 cats followed for 24 months post surgery, which had no evidence of distant metastasis at diagnosis (11). Here we propose an update of this histological staging system, inspired by the staging system applicable to human breast cancer, which takes into account the distinction between mammary carcinomas *in situ* and invasive breast cancers, measurement of the pathologic tumor size (pT), and histological detection of nodal metastases (pN, pathologic nodal stage)^2^.

Mammary carcinomas *in situ* are defined as malignant epithelial tumors that “have not extended through the basement membrane into the surrounding mammary tissue” (12). In human, they include ductal carcinomas *in situ* (DCIS) that represent 85–90% of human mammary carcinomas *in situ* (corresponding to 20% of all breast cancers), and lobular carcinomas *in situ* (LCIS) that represent 10–15% of mammary carcinomas *in situ* (0.5–3.8% of all breast cancers) (13–16)^3^. In cats, the relative frequency of mammary carcinomas *in situ* can be estimated between 1.6 and 18.8% of mammary carcinomas included in published series (11, 17–21). Two histological subtypes in particular, described by Zappulli et al. feline ductal carcinoma, and feline intraductal papillary carcinoma (22), correspond to mammary carcinomas *in situ* (surrounded by a monolayer of myoepithelial cells). There have also been descriptions of infraclinical mammary ductal carcinomas *in situ* that were adjacent to an excised mammary tumor in cats; in the series described by Burrai et al. 28/203 cats (14%) had an asymptomatic ductal carcinoma *in situ* identified at histological examination of the mammary tumor that motivated mastectomy (23). The main challenge in detecting mammary carcinomas *in situ* lies in the characterization of a continuous myoepithelial layer that encircles the carcinoma. This may be accomplished using immunochemistry to myoepithelial cell markers, including p63, calponin, CD10, cytokeratin 5 (CK5) or alpha smooth muscle actin (24–26). Absence of invasiveness is associated with good prognosis in women with breast cancer; indeed, mammary carcinomas *in situ* are rarely symptomatic (10% of cases) (13, 14), are associated with a >98% 10-year survival rate (16), and with a very low metastatic rate (<7% of patients within 15 years post-diagnosis) (27). In fact, the major risk associated with mammary ductal carcinomas *in situ* in women is ipsilateral or contralateral, *in situ* or invasive local recurrence (27–30): between 14–53% of DCIS may progress to invasive cancer over a period of 10 or more years (31).

The clinical tumor size of feline mammary carcinomas is recognized as an unfavorable prognostic factor associated with poor survival (7–10, 32, 33). The term “pathologic tumor size” was then introduced by Zappulli et al. (34). The pathologic tumor size (pT) is defined as the largest diameter of the mammary carcinoma measured in millimeters by the pathologist before paraffin embedding or on histological slides. At 20 mm threshold, pT shows a strong prognostic value in terms of overall survival, both in univariate and multivariate analyses with the histological grade and pathologic nodal stage as covariates (35).

The pathologic nodal stage (pN) characterizes the presence/absence of metastases in the regional lymph node evaluated by the pathologist using histology and immunohistochemistry. The term “nodal metastases” in breast cancer covers a range of sub-categories according to the size of metastatic deposits (macrometastases >2 mm, micrometastases 0.2–2 mm, and isolated tumor cells <0.2 mm or 200 cells), the number of axillary lymph nodes involved (1–3, 4–9, ≥10), and the location of involved lymph nodes (level I–III axillary or supraclavicular lymph nodes)^2^. In women, nodal metastases are observed of 24–33% of patients with invasive carcinomas (36–41). Nodal metastases of breast cancers are associated with poor outcomes in terms of recurrence, metastases and survival, particularly if they correspond to macrometastases and affect a high number of lymph nodes (42–45). In cats, a positive pathologic nodal stage is observed in 25% of cats with invasive carcinomas and is associated with reduced overall survival time (9).

In feline medicine, the draining lymph node of a mammary carcinoma is unfortunately not systematically removed during mastectomy, thus the pathologic nodal stage is not always available. In order to assess regional tumor spread, lymphovascular invasion (LVI) can be used as a surrogate for pN, since the presence of tumor emboli in lymphatics is strongly associated with nodal metastases (46). LVI can be detected on hematoxylin-eosin stained slides, however a better sensitivity is achieved with immunohistochemical markers of lymphatic endothelium, i.e., LYVE-1 (47, 48), Prox-1 (48), and particularly D2-40 in breast cancer (48, 49). In women, LVI is observed in 25–33% of patients with invasive mammary carcinomas (50) and is associated with a poor prognosis in terms of locoregional recurrence (51–54), time to distant metastases (55, 56), overall survival (51, 57), and cancer-specific survival (55, 58). As a matter of fact, LVI is such a strong prognostic factor in human breast cancer that Rakha et al. concluded, “LVI should be incorporated into breast cancer staging systems” (55). In cats, lymphovascular invasion has been described in 26–53% of cats with mammary carcinomas and significantly associated with reduced overall survival (8, 10, 33, 59, 60). Because the D2-40 antibody does not cross-react in cats, immunohistochemical improvement of LVI detection in feline mammary carcinomas has been achieved using other markers, i.e., von Willebrand factor (8) or LMO2 (LIM domain only 2), a transcription factor whose primary interest lies in diffuse large B-cell lymphoma classification, but which is a lymphatic endothelial marker in cats (35).

Combinations of the abovementioned parameters in staging systems have shown their prognostic value, both in women with breast cancer (invasiveness, pT and pN),^2^ and in cats with mammary carcinoma (invasiveness, pN, LVI) (11), with poorer prognosis for patients with higher stages (11)^4^. In Part 1 of this article, we proposed a histological staging system for dogs with mammary carcinoma, which combines these 4 parameters (invasiveness, pT, pN, and LVI), and was significantly associated with disease-free interval, overall survival, and specific survival.

The objectives of this part 2 were (1) to apply the histological staging system introduced in dogs (Part 1) to FMCs, and (2) to validate its prognostic value in terms of patient outcomes (disease-free interval, overall survival, and specific survival).

## Materials and Methods

### Patients and Follow-Up

This retrospective study included 395 female cats diagnosed with mammary carcinoma between 2007 and 2010, of which 340 with stage I–III invasive mammary carcinoma have been previously described (35), and 55 had a mammary carcinoma *in situ*. The owners' written consent and approval from the local Animal Welfare Committee of Oniris (College of Veterinary Medicine, Food Science and Engineering, Nantes, France) were obtained prior to inclusion. Inclusion criteria, exclusion criteria, and outcome parameters (disease-free interval, overall survival, and specific survival) were as described in dogs (Part 1).

### Histopathology and Immunohistochemistry

Histological examination was performed on 3-μm-thick hematoxylin–eosin-saffron (HES) stained whole sections (not partial biopsies) of feline mammary carcinomas. Histological types, histological grades according to Elston & Ellis' criteria (61), lymphovascular invasion (LVI), local invasion of dermis or muscle, tumor-associated inflammation, central necrosis, ulceration, squamous differentiation, margin status, and pathologic nodal stage (pN) were evaluated as previously described (35, 62). The pathologic tumor size (pT) was measured as described for dogs in Part 1 of this article.

Immunohistochemistry (IHC) was performed using a Benchmark XT automated instrument (Ventana Medical Systems, Roche Diagnostics) as described in Part 1 and a previous study (62), using antibodies to p63 (monoclonal mouse anti-p63 antibody, clone 4A4, abcam ab735, 1:100), pancytokeratin (mouse monoclonal, clones AE1/AE3, Dako, 1:200), Estrogen Receptor alpha (ER, mouse monoclonal, clone C311, Santa Cruz Biotechnology, dilution 1:50), Progesterone Receptor (PR, mouse monoclonal, clone 10A9, Meridian Life Science, 1:50), Human Epidermal growth factor Receptor Type 2 (HER2, rabbit monoclonal, clone 4B5, Roche Diagnostics, prediluted), and Ki-67 (mouse monoclonal, clone MIB1, Dako, dilution 1:50). For invasive mammary carcinomas, IHC to cytokeratins 5 and 6 (CK5/6, mouse monoclonal, clone D5/16B4, Dako, dilution 1:50), cytokeratin 14 (mouse monoclonal, clone LL002, Santa Cruz Biotechnology, dilution 1:100), Epidermal Growth Factor Receptor Type 1 (EGFR, rabbit monoclonal, clone 5B7, Roche Diagnostics, prediluted), and LMO2, which in cats is a lymphatic endothelial marker and helped in LVI assessment (LIM domain-only protein-2, clone SP51, Spring M351, 1:150) were also performed (35).

Two veterinary pathologists and 1 medical pathologist examined the HES and IHC slides blindly. In case of discrepancy, cases were collectively reviewed in order to achieve a consensual diagnosis, grade, and immunohistochemical scoring.

### Histological Staging System

The histological stages were defined as in dogs (Part 1): stage 0 (mammary carcinomas *in situ*, surrounded by a continuous layer of p63+ myoepithelial cells by immunohistochemistry), stage I (invasive, pathologic tumor size ≤20 mm (pT1) with a negative or unknown nodal status (pN0–pNX) and without lymphovascular invasion, LVI–), stage II (invasive, pathologic tumor size >20 mm (pT2), pN0–pNX nodal status, and LVI–), stage IIIA (invasive, pT1, with a positive nodal stage (pN+) and/or presence of lymphovascular invasion), and stage IIIB (invasive, pT >20 mm (pT2), LVI+ and/or pN+).

### Statistical Analyses

The MedCalc® statistical software (Ostend, Belgium) was used. Continuous variables are expressed as median, range, mean ± standard deviation. Correlations between categorical variables were analyzed using the Pearson Chi^2^ test. For univariate survival analyses (Kaplan–Meier curves and log-rank tests) and multivariate survival analyses (Cox proportional hazards models), reported results include the Hazard Ratio (HR), its confidence interval (95% CI), and the *p*-value of each covariate. For all statistical tests, a *p*-value < 0.05 was considered significant.

## Results

### Patients Characteristics

Three hundred and ninety-five queens fulfilled the inclusion criteria (Table 1).

**Table 1:** Baseline characteristics of cats.

**Parameter**		**Total**	**Stage 0**	**Stage I**	**Stage II**	**Stage IIIA**	**Stage IIIB**	***p*-value**
		***N* = 395**	***N* = 55**	***N* = 103**	***N* = 56**	***N* = 83**	***N* = 98**	
Age (years)	mean ± SD	11.1 ± 2.8	10.2 ± 3.2	11.1± 2.7	11.6 ± 2.7	11.0 ±2.6	11.5 ± 2.9	NS 0.050^a^
Hormonal status	Intact female	208 (52.7%)	31 (56.4%)	51 (49.5%)	33 (58.9%)	42 (50.6%)	51 (52.0%)	NS 0.782^b^
	Neutered female	187 (47.3%)	24 (43.6%)	52 (50.5%)	23 (41.1%)	41 (49.4%)	47 (48.0%)	
Contraception	No or Unknown	238 (60.3%)	36 (65.5%)	64 (62.1%)	32 (57.1%)	53 (63.9%)	53 (54.1%)	NS 0.556^c^
	Yes	157 (39.7%)	19 (34.5%)	39 (37.9%)	24 (42.9%)	30 (36.1%)	45 (45.9%)	
Multicentricity	Yes	61 (15.4%)	12 (21.8%)	14 (13.6%)	6 (10.7%)	10 (12.0%)	19 (19.4%)	NS 0.304^b^
	No	334 (84.6%)	43 (78.2%)	89 (86.4%)	50 (89.3%)	73 (88.0%)	79 (80.6%)	
Location^1^	M1–M2	136 (38.0%)	25 (50.0%)	31 (33.3%)	26 (47.3%)	26 (34.7%)	28 (32.9%)	NS 0.285^c^
	M3–M4	197 (55.0%)	23 (46.0%)	55 (59.1%)	25 (45.5%)	45 (60.0%)	49 (57.6%)	
	Thoraco-abdominal	25 (7.0%)	2 (4.0%)	7 (7.5%)	4 (7.3%)	4 (5.3%)	8 (9.4%)	
Surgical treatment	Nodulectomy	30 (7.6%)	5 (9.1%)	8 (7.8%)	10 (17.9%)	2 (2.4%)	5 (5.1%)	0.0001^b^
	Single mastectomy	99 (25.1%)	26 (47.3%)	23 (22.3%)	17 (30.3%)	14 (16.9%)	19 (19.4%)	
	Regional mastectomy	69 (17.5%)	6 (10.9%)	19 (18.4%)	4 (7.1%)	19 (22.9%)	21 (21.4%)	
	Unilateral radical mastectomy	188 (47.6%)	18 (32.7%)	50 (48.5%)	24 (42.9%)	46 (%)	50 (51.0%)	
	Bilateral radical mastectomy	9 (2.3%)	0	3 (2.9%)	1 (1.8%)	2 (%)	3 (3.1%)	
Inflammation	Moderate to severe	187 (47.3%)	12 (21.8%)	38 (36.9%)	28 (50.0%)	49 (59.0%)	60 (61.2%)	<0.0001^b^
	Absent to mild	208 (52.7%)	43 (78.2%)	65 (63.1%)	28 (50.0%)	34 (41.0%)	38 (38.8%)	
Central necrosis	Yes	340 (86.1%)	34 (61.8%)	88 (85.4%)	55 (98.2%)	72 (86.7%)	91 (92.9%)	<0.0001^c^
	No	55 (13.9%)	21 (38.2%)	15 (14.6%)	1 (1.8%)	11 (13.3%)	7 (7.1%)	
Margins	Negative	215 (54.4%)	46 (83.6%)	71 (68.9%)	27 (48.2%)	41 (49.4%)	30 (30.6%)	<0.0001^b^
	Positive	180 (45.6%)	9 (16.4%)	32 (31.1%)	29 (51.8%)	42 (50.6%)	68 (69.4%)	
Histological grade	I	44 (11.1%)	34 (61.8%)	7 (6.8%)	1 (1.7%)	1 (1.2%)	1 (1.0%)	<0.0001^c^
	II	189 (47.8%)	19 (34.5%)	54 (52.4%)	29 (51.8%)	43 (51.8%)	44 (44.9%)	
	III	162 (41.1%)	2 (3.6%)	42 (40.8%)	26 (46.5%)	39 (47.0%)	53 (54.1%)	
ER	mean index (%) ± SD	8.4 ± 11.5	6.4 ± 9.5	8.0 ± 11.8	8.0 ± 8.7	9.3 ± 12.3	9.3 ± 13.0	NS 0.568^a^
	ER– (<10%)	295 (74.7%)	44 (80.0%)	76 (73.8%)	43 (76.8%)	60 (72.3%)	72 (73.5%)	NS 0.857^b^
	ER+ (≥10%)	100 (25.3%)	11 (20.0%)	27 (26.2%)	13 (23.2%)	23 (27.7%)	26 (26.5%)	
PR	mean index (%) ± SD	3.2 ± 10.1	11.0 ± 14.6	3.3 ± 10.8	1.2 ± 3.5	2.6 ± 11.8	0.3 ± 0.9	<0.0001^a^
	PR– (<10%)	358 (90.6%)	33 (60.0%)	94 (91.3%)	54 (96.4%)	79 (95.2%)	98 (100%)	<0.0001^c^
	PR+ (≥10%)	37 (9.4%)	22 (40.0%)	9 (8.7%)	2 (3.6%)	4 (4.8%)	0 (0%)	
HER2	Score 0	227 (57.4%)	28 (50.9%)	65 (63.1%)	29 (51.8%)	47 (56.6%)	58 (59.2%)	NS 0.258^b^
	Score 1+	130 (32.9%)	18 (32.7%)	30 (29.1%)	21 (37.5%)	25 (30.1%)	36 (36.7%)	
	Score 2+	38 (9.7%)	9 (16.4%)	8 (7.8%)	6 (10.7%)	11 (13.3%)	4 (4.1%)	
Immunophenotype	Luminal	128 (32.4%)	29 (52.7%)	33 (32.0%)	15 (26.8%)	25 (30.1%)	26 (26.5%)	0.012^b^
	Triple-negative	267 (67.6%)	26 (47.3%)	70 (68.0%)	41 (73.2%)	58 (69.9%)	72 (73.5%)	
Ki-67	mean index (%) ± SD	44.1 ± 16.0	25.0 ± 9.8	46.0 ± 14.7	52.5 ± 15.2	44.2 ± 13.1	47.8 ± 14.6	<0.0001^a^
	Ki-67 <20%	30 (7.6%)	18 (32.7%)	5 (4.9%)	2 (3.6%)	3 (3.6%)	2 (2.0%)	<0.0001^c^
	Ki-67 ≥20%	365 (92.4%)	37 (67.3%)	98 (95.1%)	54 (96.4%)	80 (96.4%)	96 (98.0%)	
CK5/6^2^	CK5/6– (<1%)	126 (37.1%)	undetermined	39 (37.9%)	17 (30.4%)	34 (41.0%)	36 (36.7%)	NS 0.647^b^
	CK5/6+ (≥1%)	214 (62.9%)	undetermined	64 (62.1%)	39 (69.6%)	49 (59.0%)	62 (63.3%)	
CK14^2^	CK14– (<15%)	75 (22.1%)	undetermined	26 (25.2%)	3 (5.4%)	19 (22.9%)	27 (27.6%)	0.009^c^
	CK14+ (≥15%)	265 (77.9%)	undetermined	77 (74.8%)	53 (94.6%)	64 (77.1%)	71 (72.4%)	
EGFR^2^	EGFR– (<10%)	41 (12.1%)	undetermined	17 (16.5%)	5 (8.9%)	9 (10.8%)	10 (10.2%)	NS 0.412^b^
	EGFR+ (≥10%)	299 (87.9%)	undetermined	86 (83.5%)	51 (91.1%)	74 (89.2%)	88 (89.8%)	

a Analysis of variance.

b Chi-square test.

c Fisher's exact test.

1 On a total of 358 cases (37 others from unknown location).

2 Only available for stage I–IIIB (invasive) carcinomas (N = 340).

NS, Not Significant.

*SD, Standard Deviation*.

The 395 cats included 346 European (Shorthair or Longhair) cats (87.6%), 19 Siamese cats (4.8%), 7 Persian cats (1.8%), and 23 cats of other pure breeds or cross-bred (5.8%). The mean pathologic tumor size was 17 ± 8 mm (median 17 mm, range 2–48 mm, *N* = 343 cats); in the other 52 cases, the pathologic tumor size could not be precisely determined due to larger size and/or positive margins. At 20 mm threshold, 164 cats (41.5%) had a tumor larger than 20 mm in diameter (pT2). There were 168 FMCs (43%) with lymphovascular invasion, and 227 (57%) without. Nodal stage was pN+ (with metastasis of any size) in 97 cases (24.6%), pNX in 259 cats (65.6%), and pN0 in 39 cats (9.9%). The presence of distant metastases at diagnosis (M1) was an exclusion criterion in this study; there were 141 M0 cases (35.7%, no distant metastases at diagnosis), and 254 MX cases (64.3%, undetermined distant metastasis status).

Although the Veterinary Society of Surgical Oncology (VSSO) recommends that feline mammary tumors should be surgically managed by “radical mastectomy regardless of tumor size, with en bloc removal of adhered tissue due to invasive nature,”^5^ the surgical procedures performed in cats of the present series were nodulectomy in 30 cats (7.6%), single mastectomy in 99 cats (25.1%), regional mastectomy in 69 cats (17.5%), unilateral radical mastectomy in 188 cats (47.6%), and bilateral radical mastectomy in only 9 cats (2.3%). Margins were positive in 180/395 cats (45.6%), indicative of incomplete surgical resection of tumor tissue. There was no significant association between surgical procedure and margins status (*p* = 0.147).

The predominant histological types were cribriform (*N* = 184; 46.6%), solid (*N* = 68; 17.2%), tubulopapillary (*N* = 45, 11.4%), mucinous (*N* = 37, 9.4%), tubular (*N* = 28, 7.1%), and papillary (*N* = 22, 5.6%). The mean mitotic index was 48 ± 32 mitoses in 10 high-power fields (×400, diameter of the field of view 0.625 mm; median 41, range 1–164 mitoses).

One hundred FMCs (25.3%) were ER-positive, and 37 (9.4%) were PR-positive. HER2 was scored 0 in 227 FMCs (57.4%), 1+ in 130 (32.9%) cases, and 2+ in the other 38 (9.7%): this cohort did not contain any HER2-positive cases. Thus, 128 FMCs (32.4%) were luminal, and 267 (67.6%) were triple-negative. Among invasive carcinomas (*N* = 340), 214 (62.9%) were CK5/6-positive, 265 (77.9%) were CK14-positive, and 299 (87.9%) were EGFR-positive.

### Histological Stage Criteria

**Figure 1:**
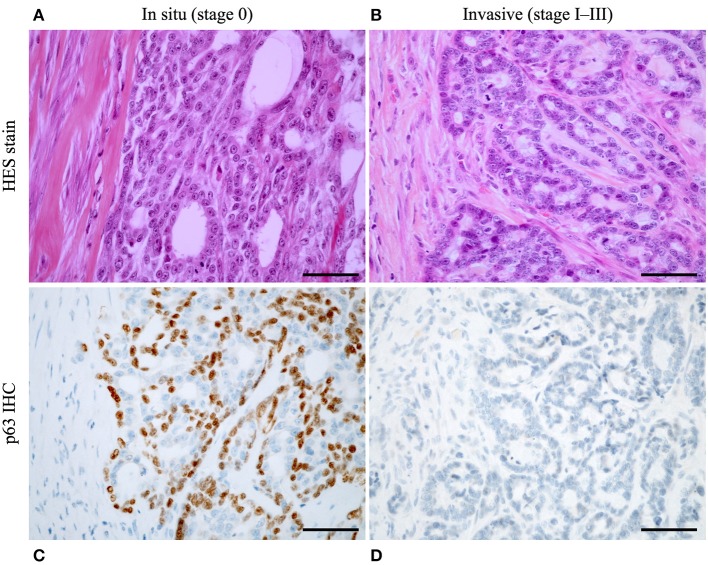
Discrimination between stage 0 (*in situ*) feline mammary carcinomas and stage I–III (invasive) FMCs using p63 immunohistochemistry. **(A)** Mammary carcinoma *in situ*, Hematoxylin-Eosin-Saffron stain. Example of a tubular carcinoma. **(B)** Invasive mammary carcinoma, HES stain. Example of a tubular carcinoma. **(C)** Same case as **(A)**, presence of a continuous layer of hypertrophic myoepithelial cells surrounding the neoplastic cells and showing strong nuclear p63 immunoreactivity. **(D)** Same case as **(B)**, absence of p63+ myoepithelial cells surrounding the neoplastic cells. HES stain **(A,B)** and P63 immunohistochemistry **(C,D)**, original magnification 400x, scale bar = 50 micrometers.

As described in dogs (Part 1), the histological stages were defined using 4 criteria: local invasiveness (identification of FMCs *in situ* by p63 IHC, Figure 1), the pathologic tumor size (pT), pathologic nodal stage (pN), and lymphovascular invasion. The four components of the proposed histological staging system were all significantly associated with disease-free interval, overall survival, and cancer-specific survival, by univariate analyses (Table 2, upper lines). However, by multivariate analyses, the DFI significantly depended only on invasiveness and lymphovascular invasion, whereas the pathologic tumor size and nodal stage were not significantly informative (Table 2, lower lines, model 1). For overall and cancer-specific survival assessment, the pathologic nodal stage was not a significantly independent prognostic factor. This could be explained by the strong correlations exiting between pN and pT (Odds ratio 2.15, 95% CI 1.35–3.42, *p* = 0.0017), between pN and LVI (Odds ratio 16.46, 95% CI 8.71–31.10, *p* < 0.0001), and between pN and invasiveness (*p* < 0.0001, none of the pN+ FMCs were *in situ*) in the present cohort, explaining why the prognostic value of pN was not significantly independent by multivariate analyses, although it was highly significant by univariate analysis.

**Table 2:** Prognostic value of the parameters included in the histological staging system.

**Univariate analyses**		**Disease-free interval**		**Overall survival**		**Cancer-specific survival**	
		**HR (95% CI)**	***P***	**HR (95% CI)**	***P***	**HR (95% CI)**	***P***
Invasiveness	Invasive vs. *in situ*	3.15 (2.34–4.24)	<0.0001	2.60 (2.02–3.35)	<0.0001	2.79 (2.07–3.77)	<0.0001
Pathologic tumor size	>20 vs. ≤ 20 mm	1.54 (1.17–2.03)	0.0010	1.82 (1.45–2.29)	<0.0001	1.84 (1.41–2.40)	<0.0001
Lymphovascular invasion	LVI+ vs. LVI–	2.01 (1.52–2.67)	<0.0001	2.19 (1.73–2.77)	<0.0001	2.46 (1.87–3.23)	<0.0001
Pathologic nodal stage	pN+ vs. pN0–PNX	1.68 (1.19–2.36)	0.0004	1.84 (1.39–2.44)	<0.0001	2.08 (1.50–2.88)	<0.0001
LVI × pN	LVI+ and/or pN+ vs. LVI– pN0,pNX	1.89 (1.44-2.49)	<0.0001	2.09 (1.67–2.63)	<0.0001	2.31 (1.78–3.01)	<0.0001
**Multivariate analyses Model 1**							
Invasiveness	Invasive vs. *in situ*	2.69 (1.68–4.30)	<0.0001	1.95 (1.34–2.83)	0.0005	1.94 (1.23–3.07)	0.0047
Pathologic tumor size	>20 vs. ≤20 mm	1.28 (0.98–1.68)	0.0727	1.53 (1.23–1.91)	0.0002	1.50 (1.16–1.94)	0.0021
Lymphovascular invasion	LVI+ vs. LVI–	1.48 (1.09–2.01)	0.0118	1.63 (1.26–2.11)	0.0002	1.79 (1.32–2.41)	0.0002
Pathologic nodal stage	pN+ vs. pN0–PNX	1.12 (0.81–1.56)	0.4990	1.18 (0.90–1.55)	0.2391	1.29 (0.94–1.76)	0.1133
**Model 2**							
Invasiveness	Invasive vs. *in situ*	2.74 (1.71–4.40)	<0.0001	1.96 (1.34–2.85)	0.0005	1.97 (1.24–3.13)	0.0040
Pathologic tumor size	>20 vs. ≤20 mm	1.31 (1.00–1.71)	0.0445	1.57 (1.26–1.96)	<0.0001	1.56 (1.21–2.01)	0.0006
LVI × pN	LVI+ and/or pN+ vs. LVI– pN0,pNX	1.43 (1.09–1.88)	0.0104	1.66 (1.32–2.09)	<0.0001	1.85 (1.42–2.43)	<0.0001

Then we combined the parameters LVI and pN, and this “LVI × pN” parameter was significantly associated with disease-free survival, overall survival, and cancer-specific survival, both in univariate analysis (Table 2, upper lines), and multivariate analyses (Table 2, lower lines, model 2), independently of invasiveness and tumor size. Thus, the proposed staging system for cats with FMC relies on 3 parameters (invasiveness, pT, LVI × pN) that were all significantly informative in outcome assessment.

### Differences in Initial Presentation According to Histological Stages

At initial presentation, mammary carcinomas *in situ* (stage 0, *N* = 55) significantly differed from invasive (stage I–III, *N* = 340) FMCs (Table 3). Briefly, stage 0 FMCs were diagnosed at younger age, were smaller, of lower histological grade, with less common tumor-associated inflammation and central necrosis, more common negative margins, higher PR expression, and a lower Ki-67 proliferation index, than invasive FMCs.

**Table 3:** Main differences between stage 0 (*in situ*) and stage I–III (invasive) feline mammary carcinomas.

**Parameter**		**Stage 0*N* = 55**	**Stage I–III*N* = 340**	***p*-value**
Age at diagnosis (years)	Mean ± SD	10.2 ± 3.2	11.3 ± 2.7	0.010
	Median	10.3	11.1	
	Range	2.8–17.3	4.0–21.3	
Pathologic tumor size (mm)	Mean ± SD	12 ± 7	18 ± 7	<0.001
	Median	11	17	
	Range	2–32	3–48	
Histological grade	I	34 (61.8%)	10 (2.9%)	<0.0001
	II	19 (34.5%)	170 (50.0%)	
	III	2 (3.6%)	160 (47.1%)	
Tumor-associated inflammation	Moderate to severe	12 (21.8%)	175 (51.5%)	0.0001
	Absent to mild	43 (78.2%)	165 (48.5%)	
Central necrosis	Yes	34 (61.8%)	306 (90.0%)	<0.0001
	No	21 (38.2%)	34 (10.0%)	
Margins	Negative	46 (83.6%)	169 (49.7%)	<0.0001
	Positive	9 (16.4%)	171 (50.3%)	
PR	Mean index (%) ± SD	11.0 ± 14.6	1.9 ± 8.5	<0.001
	PR– (<10%)	33 (60.0%)	325 (95.6%)	<0.0001
	PR+ (≥10%)	22 (40.0%)	15 (4.4%)	
Ki-67	Mean index (%) ± SD	25 ± 10	47 ± 15	<0.001
	Median	25	46	

There were also significant differences in initial presentation according to histological stages of invasive FMCs (Table 1). Stage IIIA–IIIB FMCs were more commonly diagnosed with positive margins (110/181, 61%) than stage I–II FMCs (61/159, 38%, *p* = 0.0001). Compared with stage I–II FMCs, stage IIIA-IIIB FMCs were more commonly associated with moderate to severe peritumoral inflammation (in 109/181 (60%) stage IIIA-IIIB FMCs vs. 66/159 (42%) stage I–II FMCs, *p* = 0.0009). The pathologic tumor size was significantly higher in stage IIIA–IIIB FMCs (mean 20 ± 7 mm, median 19 mm) than in stage I–II FMCs (mean 16 ± 8 mm, median 15 mm, *p* < 0.001). However, there were no significant differences in age at diagnosis, histological grade, ER and PR expression, Ki-67 proliferation index, and basal marker expression (CK5/6, CK14, EGFR) between stage I–II FMCs and stage IIIA–IIIB FMCs.

### Disease-Free Interval by Histological Stage

During the follow-up period, 106/395 cases recurred locally (27%), including 98/340 invasive FMCs (29%) and 8/55 mammary carcinomas *in situ* (stage 0 FMCs, 15%), of which 29 invasive FMCs (30%) and 4 stage 0 FMCs (50%) were subjected to a second surgery. Nodal metastasis was diagnosed during the follow-up period in 11/395 cats (3%), all of which with invasive (stage I–III) FMCs. Distant metastasis occurred in 133/395 cats (34%), including 117/340 cats with invasive (stage I–III) FMCs (34%), and 16/55 cats with stage 0 (*in situ*) FMCs (29%).

The median DFI was 438 days (1 year and 2.4 months). Cancer progression (locoregional recurrence and/or distant metastasis) was recorded in 49% of cats at 1-year post diagnosis, and 61% at 2 years.

**Figure 2:**
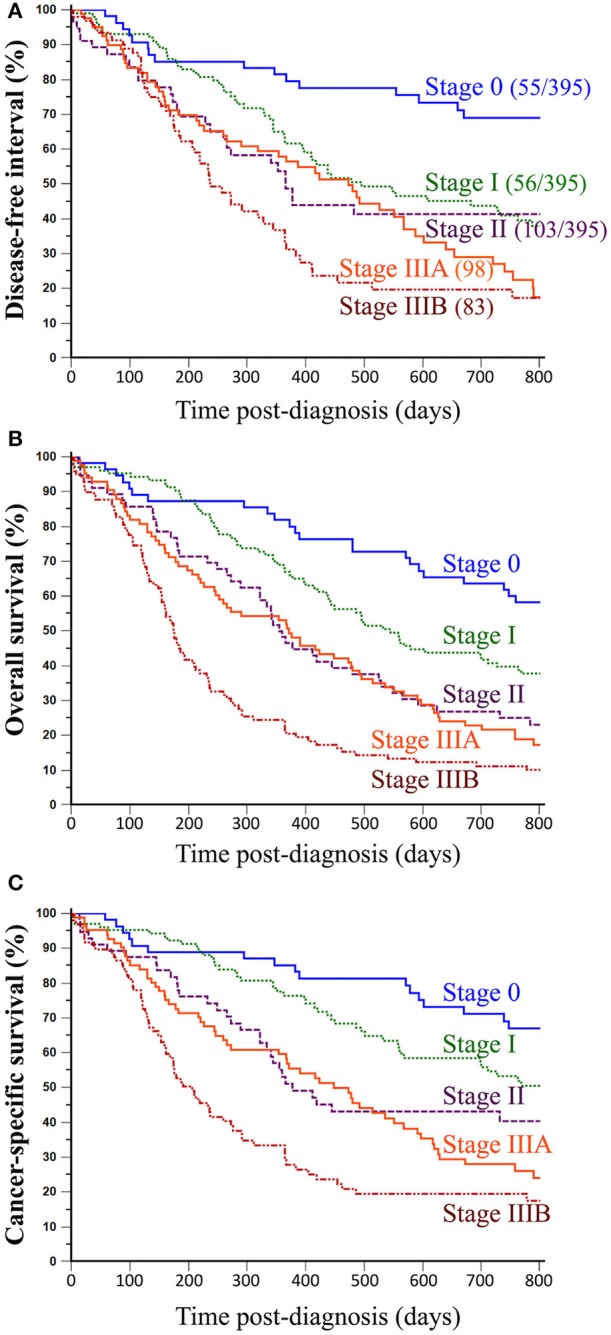
Association between histological stages of FMCs and outcomes of feline patients. **(A)** Disease-free interval. The probability of locoregional recurrence and/or distant metastasis was very low for stage 0 FMCs, moderate for stage I–II FMCs, and high for stage IIIA–IIIB FMCs. **(B)** Overall survival. All-cause mortality of female cats with mammary carcinoma significantly increased with increasing histological stage at presentation, although there was poor separation between stage II and stage IIIA FMCs. **(C)** Cancer-specific survival. The probability of dying from cancer significantly increased with histological stage. Kaplan-Meier curves. See Table 4 for corresponding hazard ratios and *p*-values.

When split by histological stage, median disease-free intervals were 1484 days (4 years and 0.8 months) for stage 0 FMCs, 496 days (1 year and 4.3 months) for stage I FMCs, 366 days (1 year) for stage II FMCs, 473 days (1 year and 3.5 months) for stage IIIA FMCs, and 237 days (7.8 months) for stage IIIB FMCs. However, there was no clear separation between stage II and stage IIIA FMCs (Figure 2A). The probabilities of cancer progression within 1 year post diagnosis were 19% for stage 0, 36% for stage I, 46% for stage II, 42% for stage IIIA, and 67% for stage IIIB FMCs. Compared to stage 0 FMCs (HR = 1.00, reference), the probabilities of cancer progression were 2.59 times higher for stage I FMCs (*p* = 0.0002), 3.42 times higher for stage II FMCs (*p* < 0.0001), 3.66 times higher for stage IIIA FMCs (*p* < 0.0001), and 4.99 times higher for stage IIIB FMCs (*p* < 0.0001; Table 4 and Figure 2A).

**Table 4:** Prognostic factors of feline mammary carcinomas.

**Univariate analyses**		**Disease-free interval**		**Overall survival**		**Cancer-specific survival**	
		**HR (95% CI)**	***P***	**HR (95% CI)**	***P***	**HR (95% CI)**	***P***
Margin status	Positive vs. negative	1.40 (1.07–1.82)	0.0100	1.68 (1.35–2.10)	<0.0001	1.63 (1.26–2.11)	0.0001
Tumor-associated inflammation	Moderate to severe vs. absent to mild	1.32 (1.02–1.72)	0.0306	1.40 (1.13–1.74)	0.0016	1.55 (1.20–1.99)	0.0005
Dermal invasion	Yes vs. no	1.76 (1.35–2.27)	<0.0001	2.06 (1.67–2.57)	<0.0001	2.26 (1.76–2.91)	<0.0001
Cutaneous ulceration	Yes vs. no	–	NS	1.94 (1.40–2.69)	<0.0001	1.78 (1.23–2.59)	0.0002
Histological grade	III vs. I	2.77 (1.68–4.56)	0.0001	2.64 (1.79–3.89)	<0.0001	3.27 (1.96–5.47)	<0.0001
	II vs. I	2.45 (1.51–4.00)	0.0003	1.81 (1.23–2.65)	0.0027	2.53 (1.52–4.19)	0.0004
ER	ER+ vs. ER–	1.44 (1.05–1.98)	0.0109	–	NS	1.34 (0.99–1.80)	0.0381
PR	PR+ vs. PR–	0.41 (0.29–0.59)	0.0003	0.52 (0.39–0.71)	0.0008	0.38 (0.26–0.54)	0.0002
Ki-67	≥20% vs. <20%	2.27 (1.50–3.42)	0.0042	1.90 (1.34–2.71)	0.0053	2.62 (1.73–3.97)	0.0018
Histological stage	IIIB vs. 0	4.99 (3.03–8.24)	<0.0001	4.77 (3.21–7.07)	<0.0001	5.24 (3.26–8.44)	<0.0001
	IIIA vs. 0	3.66 (2.21–6.07)	<0.0001	2.91 (1.94–4.36)	<0.0001	3.34 (2.06–5.43)	<0.0001
	II vs. 0	3.42 (1.99–5.90)	<0.0001	2.76 (1.79–4.28)	<0.0001	2.86 (1.69–4.85)	0.0001
	I vs. 0	2.59 (1.59–4.24)	0.0002	1.80 (1.21–2.67)	0.0039	1.78 (1.10–2.90)	0.0204

*NS, Not Significant*.

By univariate analysis, 7 parameters other than histological stage were significantly associated with disease-free interval (Table 4), i.e., margin status, tumor-associated inflammation, dermal invasion, the histological grade, ER and PR expression and the Ki-67 proliferation index.

In cats with invasive FMC, none of the above parameters were associated with DFI independently of the histological stage by multivariate analysis: the proposed histological stage was the strongest prognostic factor for DFI assessment, and no other clinico-pathologic criteria added significant prognostic information.

### Overall Survival by Histological Stage

During the follow-up period, 339 cats (85.8%) died. The median overall survival time was 377 days (1 year and 0.4 month; range, 2 days−2195 days). The mortality rate was 47% at 1 year and 70% at 2 years post diagnosis. Death was unrelated to cancer in 32 cats (8.1%), from unknown causes in 58 cats (14.7%), and attributable to the mammary carcinoma in 249 cats (63.0%).

The proposed histological stages showed significant association with all-cause mortality of female cats of the present study. Indeed, median overall survival times were 999 days for stage 0 FMCs (2 years and 8.8 months), 545 days for stage I FMCs (1 year and 5.9 months), 355 days for stage II FMCs (11.6 months), 372 days for stage IIIA FMCs (12.2 months), and 175 days for stage IIIB FMCs (5.7 months). As for DFI, there was very poor separation between stage II and stage IIIA FMCs (Figure 2B). The probabilities of death from all causes within 1 year post diagnosis were 18% for stage 0, 33% for stage I, 52% for stage II, 47% for stage IIIA, and 79% for stage IIIB FMCs. Compared to stage 0 FMCs (HR = 1.00, reference), the probabilities of dying from any cause were 1.80 times higher for stage I FMCs (*p* = 0.0039), 2.76 times higher for stage II FMCs (*p* < 0.0001), 2.91 times higher for stage IIIA FMCs (*p* < 0.0001), and 4.77 times higher for stage IIIB FMCs (*p* < 0.0001; Table 4 and Figure 2B).

Apart from histological stage, 7 other parameters were significantly associated with overall survival (Table 4), i.e., margin status, tumor-associated inflammation, dermal invasion, cutaneous ulceration, the histological grade, PR expression, and the Ki-67 proliferation index.

In the 340 patients with invasive FMCs, the histological stage (HR = 2.45 for stage IIIB, HR = 1.68 for stage IIIA FMCs compared to stage I) was a significant predictor of overall survival by multivariate analysis (Table 5), with 2 independent covariates (*p* < 0.0001): the histological grade (HR = 1.27 for grade III FMCs compared to grade I–II), and cutaneous ulceration (HR = 1.46 when present).

**Table 5:** Prognostic value of the histological staging system applied to invasive mammary carcinomas.

**Multivariate analyses**		**Overall survival**		**Cancer-specific survival**	
		**HR (95% CI)**	***P***	**HR (95% CI)**	***P***
Histological stage	IIIB vs. I	2.45 (1.80–3.32)	<0.0001	2.72 (1.90–3.88)	<0.0001
	IIIA vs. I	1.68 (1.23–2.29)	0.0012	1.76 (1.22–2.54)	0.0025
	II vs. I	1.39 (0.97–1.98)	0.0719	1.50 (0.98–2.28)	0.0625
Tumor-associated inflammation	Moderate to severe vs. absent to mild	–	NS	1.33 (1.02–1.73)	0.0370
Cutaneous ulceration	Yes vs. no	1.46 (1.10–1.93)	0.0088	–	NS
Histological grade	III vs. I–II	1.27 (1.01–1.60)	0.0413	–	NS
PR	PR+ vs. PR–	–	NS	0.34 (0.13–0.92)	0.0352

*NS, Not Significant*.

### Specific Survival by Histological Stage

The median time to death attributable to cancer was 496 days (1 year and 4.3 months, range, 2–2195 days). The cancer-related death rate was 39% at 1 year and 59% at 2 years post diagnosis. These survival probabilities were highly dependent upon histological stage at diagnosis: median specific survival times were 1484 days for stage 0 FMCs (4 years and 0.8 months), 808 days for stage I FMCs (2 years and 2.6 months), 377 days for stage II FMCs (12.4 months), 448 days for stage IIIA FMCs (14.7 months), and 207 days for stage IIIB FMCs (6.8 months). As shown in Figure 2C, there was poor separation between stage II and stage IIIA FMCs within 550 days post-diagnosis, but thereafter the 4 stages were correctly separated. The probabilities of cancer-related death within 1-year post diagnosis were 15% for stage 0, 24% for stage I, 49% for stage II, 40% for stage IIIA, and 71% for stage IIIB FMCs. Compared to stage 0 FMCs (HR = 1.00, reference), the probabilities of dying from cancer were 1.78 times higher for stage I FMCs (*p* = 0.0204), 2.86 times higher for stage II FMCs (*p* = 0.0001), 3.34 times higher for stage IIIA FMCs (*p* < 0.0001), and 5.24 times higher for stage IIIB FMCs (*p* < 0.0001; Table 4 and Figure 2C).

By univariate analysis, 8 parameters other than histological stage were significantly associated with cancer-specific survival (Table 4): margin status, tumor-associated inflammation, dermal invasion, cutaneous ulceration, the histological grade, ER and PR expression, and the Ki-67 proliferation index.

In the 340 female cats with invasive mammary carcinomas, the risk of cancer-related death was predicted by 3 independent prognostic factors by multivariate analysis (p < 0.0001; Table 5): the histological stage (HR = 2.72 for stage IIIB, HR = 1.76 for Stage IIIA compared to stage I), PR expression (HR = 0.34 for PR+ FMCs compared to PR–), and tumor-associated inflammation (HR = 1.33 when moderate to severe compared to absent to mild). These results indicated that the proposed histological system, PR expression, and tumor-associated inflammation were the strongest prognostic factors associated with cancer-related death probabilities in cats with invasive mammary carcinomas.

## Discussion

This study evaluated the prognostic value of the histological staging system proposed for dogs (Part 1), in a large cohort of 395 female cats with mammary carcinomas, including 340 invasive (stage I–III), and 55 *in situ* (stage 0) mammary carcinomas. This staging system may be considered an update of the 3-stage system proposed by Preziosi et al. in 2002, which takes into account local invasiveness (distinction between non-infiltrating and invasive carcinomas), the presence of neoplastic emboli in vessels, and regional lymph node involvement (11). The 3 stages (0, I, II) defined by Preziosi et al. were significantly associated with overall survival, both in univariate and multivariate analyses, with the mitotic index and an AgNOR (Agyrophilic Nucleolar Organizer Region) proliferation index as covariates (11). However, the study comprised 33 cats only (of which 3 had mammary carcinomas *in situ*), and included neither the pathologic tumor size, nor the unknown pathologic nodal stage (pNX). Despite lack of completeness of this system, these authors were the first and unique who adapted the staging system applicable to human breast cancer in female cats with FMCs. In this context, we have applied to FMCs the histological staging system proposed for dogs (invasiveness, pT, LVI, pN), inspired by breast cancer staging (invasiveness, pT, pN),^2^ in order to determine its prognostic value in cats.

Characterization of mammary carcinomas *in situ*, and consequently definition of stage 0, is an important parameter that appears in both Preziosi's staging system for cats (11), and the American Joint Committee on Cancer staging system for breast cancer^2.^ In our cohort, mammary carcinomas *in situ* were diagnosed in 55 cats (14% of the cohort) and differed in initial presentation from the other 340 invasive FMCs. FMCs *in situ* were diagnosed at younger age (median 10.3 years) than invasive FMCs (median 11.1 years). This was also observed in dogs (Part 1): stage 0 CMCs affected younger dogs (median 10.2 years) than stage I–III CMCs (median 11.0 years). In women also, mammary carcinomas *in situ* are more common in younger women (annual incidence of 81.8 per 100,000 women aged 60–69 years) than in older women (annual incidence of 47.4 per 100,000 women >80 years) (63). This younger age at diagnosis may be due to the fact that mammary carcinomas *in situ*, particularly ductal carcinomas *in situ*, are considered as non-obligate precursors to invasive carcinomas, preceded by atypical ductal hyperplasia in the carcinogenetic continuum (28, 64). Both in cats and dogs in our studies, mammary carcinomas *in situ* were of smaller pathologic tumor size and lower histological grade than invasive FMCs, and showed a lower Ki-67 proliferation index. These results correlate with breast cancer characteristics in women: invasive breast cancers are larger than DCIS (65, 66), are of higher histological or nuclear grade (66, 67), and have a higher Ki-67 proliferation index (68). However, contrary to cats and dogs of the present study, PR expression is not significantly different in breast cancers *in situ* and invasive mammary carcinomas (66). In cats and dogs, mammary carcinomas *in situ* were associated with better disease-free interval (HR = 5.07 in dogs and 3.15 in cats), overall survival (HR = 2.86 in dogs and 2.60 in cats), and specific survival (HR = 6.78 in dogs and 2.79 in cats for invasive vs. *in situ* mammary carcinomas, *p* < 0.0001). In women, the prognosis associated with DCIS is very good also (16, 27, 69), even if mortality is increased after local invasive recurrence of DCIS (27). These similarities in behavior of mammary carcinomas *in situ* in cats, dogs and women, justify the identification of stage 0 FMCs in female cats.

The pathologic tumor size (pT) was not integrated in Preziosi's histological staging system for FMCs (11), but is involved in breast cancer staging. In the current clinical staging system used in cats, two thresholds for tumor size are defined: ≥2 and ≥3 cm (6), which differ from the thresholds of ≥3 and >5 cm used for canine mammary carcinomas (70). In the present staging system, we have chosen to use a unique threshold of >20 mm in both species, in order to facilitate tumor size assessment by the veterinary pathologist. Indeed, the pathologic tumor size in the present series was very similar for FMCs (mean 17 ± 8 mm, median 17 mm) and CMCs (mean 16 ± 7 mm, median 17 mm); also, patient repartition in the pT1 and pT2 categories was similar: 42% of FMCs and 47% of CMCs were pT2. The prognostic value associated with pT was similarly strong in dogs with CMC as in cats with FMC, both in terms of overall survival (HR = 1.82 in dogs and 1.82 in cats), and specific survival (HR = 1.81 in dogs and 1.84 in cats for pT2 vs. pT1 mammary carcinomas, *p* < 0.0001), as well as in disease-free interval (HR = 1.48 in dogs, *p* < 0.0001, and HR = 1.54 in cats, *p* = 0.0010 for pT2 vs. pT1 carcinomas). In women, T2 mammary carcinomas are associated with poorer prognosis compared with T1 carcinomas, with a 5-year overall survival rate of 91–95% for T1 and 82% for T2 breast cancers (71). There are also significant differences in disease-free survival between T1 and T2 breast cancers by univariate and multivariate analyses (72). However, in breast cancer staging, two other (pathologic) tumor size categories are defined: (p)T3 >50 mm and (p)T4 for tumors of any size extending to the chest wall and/or to the skin (dermal invasion, cutaneous ulceration). In the present study, cutaneous ulceration appeared as a significant prognostic factor of FMCs (HR = 1.94, *p* < 0.0001 for overall survival, HR = 1.78, *p* = 0.0002 for specific survival), as well as in dogs with CMCs (HR = 1.66, *p* = 0.0009 for overall survival, HR = 1.91, *p* = 0.0007 for specific survival). Cutaneous ulceration overlying FMCs has already been associated with poor survival (33), and argues in favor of including a pT4 category in a future staging system.

Lymphovascular invasion (LVI) is not included in breast cancer staging^2^, however appears in Preziosi's definitions of FMC histological stages (11). In the present study, LVI was a good predictor of nodal metastasis, with 86.6% sensitivity and 77.8% specificity, slightly better than in dogs with CMCs (85.6% sensitivity and 73.0% specificity). This can be explained by the fact that in cats with doubtful emboli on histological slides, LVI assessment was improved by immunolabeling of lymphatic endothelial cells using an anti-LMO2 antibody (35). LMO2 immunohistochemistry allowed decreasing false negative cases (large tumor emboli that totally obstructed the vessel lumen and compressed endothelial cells) as well as false positive cases (nests of neoplastic cells surrounded by artifactual retraction of connective tissue). The present study confirmed the negative prognostic value of LVI in FMCs, regarding disease-free interval, overall survival, and specific survival, as previously reported in cats with FMC by univariate analyses (10, 21, 33, 60), and multivariate analyses with the histological type (8) or the mitotic index (11) as independent covariates.

The pathologic nodal stage (pN) is included in both Preziosi's and breast cancer staging systems (11).^2^ As in dogs, the majority of regional lymph nodes have not been removed in cats with FMCs (65.6% of pNX cases in cats and 67.9% in dogs). We confirmed here the strong negative prognostic value of a pN+ status by univariate analysis, however pN lost its significant prognostic value in the multivariate model that included invasiveness, pT, LVI, and pN. This could be explained first because there were strong associations between invasiveness/pN, pT/pN and LVI/pN in the present study, and secondly because a great majority of pNX cases may be pN+, according to the high frequency of nodal metastasis reported at diagnosis of FMCs (8, 9). There was thus rationale to combine LVI and pN in a single parameter (“LVI × pN”), which appeared significantly associated with disease-free interval, overall survival, and cancer-specific survival, by multivariate analyses with invasiveness and pathologic tumor size as independent covariates. This validated inclusion of a LVI × pN parameter in the histological staging system proposed for cats, as previously done by Preziosi et al. (11).

Based on the 4 parameters invasiveness, pathologic tumor size, lymphovascular invasion, and pathologic nodal stage, we then applied the same 5-stage system as proposed for canine mammary carcinomas. By univariate analysis, this histological staging system allowed splitting the 395-cat cohort into well-separated risk groups, with increasing hazard ratios, especially for cancer-specific survival (Stage 0: HR = 1.00; Stage I: HR = 1.78; Stage II: HR = 2.86; Stage IIIA: HR = 3.34; Stage IIIB: HR = 5.24; *p* < 0.0001). However, in cats with invasive (stage I–III) FMCs, stage II (pT2, LVI–, pN0–pNX) and stage IIIA (pT1, LVI+ and/or pN+) FMCs did not significantly differ one from the other in disease-free interval and overall survival. We think that the differences in outcomes between stage II and stage IIIA FMCs would be better highlighted if pNX cases were less prevalent, i.e., if draining lymph nodes were systematically sampled for histopathology; then, a substantial number of cases currently identified as stage II (pT2, LVI–, pN0–pNX) would be reclassified as stage IIIB (pT2, LVI+ and/or pN+). Finally, we found by multivariate survival analysis that cancer-specific survival depended on the proposed histological stages, i.e., cancer spread within patients, as well as Progesterone Receptor positivity, and tumor-associated inflammation. This result suggests that there exists a (small) subgroup of PR+ FMCs which rarely cause death of feline patients, and that the inflammatory/immune response of the host is a significant actor in patient survival.

Here, we propose a 5-stage system that characterizes cancer extent within patients, and is strongly associated with patient outcomes; however, the surgical management of female cats with mammary carcinoma is another parameter known to impact patient survival (32, 73). In the present study, there was a bias due to the fact that stage 0 (*in situ*) FMCs had been preferentially removed by nodulectomy or single mastectomy, whereas stage IIIA–IIIB FMCs had been mostly removed by regional or radical mastectomy. In the present cohort, the surgical procedure was not significantly associated with the risk of local recurrence (*p* = 0.331), distant metastasis (*p* = 0.379), overall survival (*p* = 0.820), and specific survival (*p* = 0.620). There was no significant association between the surgical procedure and margins status (*p* = 0.147), indicating that larger resections did not lead to safer margins. However, a negative margin status was significantly associated with longer disease-free interval, overall survival, and cancer-specific survival, confirming that proper surgical management of FMCs favorably impacts the outcome.

As retrospective in nature, this study has important limitations. Female cats included in this cohort lived in different areas, and were followed in multiple veterinary clinics with different habits regarding feline mammary cancer management. Regarding clinical staging, clinical tumor size was rarely recorded; regional spread was almost never assessed using medical imaging or cytology, and we can suppose that the 136 cases (34%) in which lymph node status was assessed by histopathology were mostly those suspect of nodal metastasis at palpation; distant metastasis was assessed using 2- or 3-view thoracic radiographs in 140 cats (34%) or abdominal ultrasound in 6 cats (2%) only, and computed tomography scans were not performed. Thus, an undetermined number of cases were understaged. Regarding therapeutic management, we have chosen to exclude cats treated with adjuvant chemotherapy, as this may impact survival. However, the VSSO guidelines for surgical management of FMCs^5^ were not commonly followed, and this introduced undesirable heterogeneity in this retrospective cohort. It would be very informative to reevaluate the histological staging system proposed here, in an independent prospective cohort in which clinical staging and surgical management would follow recommended guidelines, in order to avoid pNX and MX cases, and to decrease the frequency of positive margins. Also, a prospective cohort would allow for standardization of pathologic tumor size assessment, with selection of the greatest tumor diameter during histological processing, even for very large mammary carcinomas.

In conclusion, the proposed 5-stage histological staging system previously described in female dogs with mammary carcinoma (invasiveness, pT, pN, LVI) is applicable in the feline species in order to assess prognosis. In the near future, a preoperative complete tumor clinical staging and treatment based on the published standard of care should be performed in order to better validate the histological staging system here proposed. We also hope that this staging system, which identifies a very-high risk group (stage IIIB, 25% of patients), will be used in randomized clinical trials evaluating adjuvant chemotherapy for female cats with invasive mammary carcinomas.

## Data Availability Statement

The datasets generated for this study are available on request to the corresponding author.

## Ethics Statement

The animal study was reviewed and approved by CERVO, Comité d'Ethique en Recherche clinique et épidémiologique Vétérinaire d'Oniris, Oniris, Nantes Atlantic College of Veterinary Medicine, Food Science and Engineering, Nantes, France. Written informed consent was obtained from the owners for the participation of their animals in this study.

## Author Contributions

FN conceptualization, project administration, supervision, and funding acquisition. FC, M-MB, FB, GV, DL, and FN format analysis, investigation, methodology, writing, review, and editing. FC and FN writing and original draft preparation.

### Conflict of Interest

The authors declare that the research was conducted in the absence of any commercial or financial relationships that could be construed as a potential conflict of interest.

## Footnotes

^1^Owen LN. Veterinary Public Health Unit & WHO Collaborating Center for Comparative Oncology. In: editor LN Owen. *TNM Classification of Tumors in Domestic Animals*. World Health Organization (1980). Available online at: https://apps.who.int/iris/handle/10665/68618

^2^American Joint Committee on Cancer. (2009). *Breast Cancer Staging, 7th Edn*. Available online at: https://cancerstaging.org/references-tools/quickreferences/Documents/BreastMedium.pdf

^3^Collins LC, Laronga C, Wong JS. *Breast Ductal Carcinoma in situ: Epidemiology, Clinical Manifestations, and Diagnosis*. (2019). Available online at: https://www.uptodate.com/contents/breast-ductal-carcinoma-in-situ-epidemiology-clinical-manifestations-and-diagnosis?topicRef=14220&source=see_link

^4^*SEER Cancer Statistics Review 1975-2010*. Available online at: https://seer.cancer.gov/archive/csr/1975_2010/results_merged/sect_04_breast.pdf

^5^Veterinary Society of Surgical Oncology. Mammary tumors, feline, treatment. https://vsso.org/mammary-tumors-feline
